# What Makes an Elite Shooter and Archer? The Critical Role of Interoceptive Attention

**DOI:** 10.3389/fpsyg.2021.666568

**Published:** 2021-06-09

**Authors:** Pengli Li, Quanyu Lu, Qiong Wu, Xinghua Liu, Yanhong Wu

**Affiliations:** ^1^School of Psychological and Cognitive Sciences, Peking University, Beijing, China; ^2^Beijing Key Laboratory of Learning and Cognition, School of Psychology, Capital Normal University, Beijing, China; ^3^Beijing Key Laboratory of Behavior and Mental Health, Peking University, Beijing, China; ^4^Key Laboratory of Machine Perception, Ministry of Education, Peking University, Beijing, China

**Keywords:** shooting, archery, interoceptive attention, breath detection task, mindfulness training

## Abstract

It is well-acknowledged that attention is important for expert performance in sports. However, the role of interoceptive attention, i.e., the attentional mechanism of awareness and conscious focus of bodily somatic and visceral signals, in self-paced and far-aiming sports remains to be explored. This study aims to investigate the relationship of expertise level and interoceptive attention ability in shooting and archery, and to examine if interoceptive attention can be improved by mindfulness training in elite athletes of shooting and archery. We tested the performance differences of 41 elite athletes from the Chinese national team of shooting and archery and 43 non-elite athletes from a provincial team in breath detection task (BDT) and dot flash detection task (DDT), which were designed to measure interoceptive and exteroceptive attention (i.e., attention toward information input of primary sensory), respectively. Furthermore, we applied mindfulness training to the 41 elite athletes for 5–8 weeks and remeasured their performances of BDT and DDT. Results showed that elite athletes outperformed non-elite athletes in BDT (but not in DDT) both in accuracy (Diff_BDT_ = 11.50%, *p* = 0.004) and in discrimination sensitivity (*d*′, Diff_BDT_ = 1.159, *p* = 0.002). Difference in accuracy and *d*′ reached significant level only in BDT (accuracy: Diff_BDT_ = −8.50%, *p* = 0.001; *d*′: Diff_BDT_ = −0.822, *p* = 0.003) before and after mindfulness training. These results indicate that elite athletes of shooting and archery (i.e., relative to non-elite athletes) can better perceive the somatic and visceral responses or changes and discriminate these signals from noises. Moreover, interoceptive attention can be improved by mindfulness training. These results have important implications for the selection and training of athletes of shooting and archery.

## Introduction

How to achieve excellent performance in sports or to become an elite athlete? In addition to professional skills, it is well-acknowledged that attention is vital for success in sports. Some researchers even claimed that there may be no other aspect of psychology that could be more important to athletic performance than attention (Moran, 2004; Abernethy et al., 2007). In general, attention refers to the ability to focus on specific stimulus or locations (Goldstein, 2011). It is an important part of cognitive functioning, which supports various activities of individuals and has many different manifestations, such as alerting, selective, sustained, and divided (Tamm et al., 2013; Reigal et al., 2020). Earlier studies have found that attention is related to other aspects of cognitive functioning, such as executive control, learning, and memory (Logue and Gould, 2014; Bialystok, 2015; Campillo et al., 2018). There are two lines of research with respect to the relationship between attention (as well as cognitive functioning) and sports. First, some studies focus on the benefits that physical activities bring on attention in childhood, adolescence, and senescence (Crespillo-Jurado et al., 2019; Xue et al., 2019; Hernández-Mendo et al., 2020). Second, studies have also explored the effect of attention on athletes of different sports with the intention to help them achieve success (Vestberg et al., 2017; Policastro et al., 2018; Hernández-Mendo et al., 2019). To enrich the discussion on the topic, in this study, we aimed to explore the relationship between specific attention ability (namely interoceptive attention) and expertise level in shooting and archery sports, and to examine if this attention ability can be improved through intervention.

Attention can be divided into exteroceptive attention, interoceptive attention, and executive control of attention according to information sources (Wang et al., 2019). Exteroceptive attention refers to attending to the external information input of primary sensory, such as visual stimulus. Interoceptive attention refers to attending to internal body signals, such as somatic and visceral signals. Executive control of attention refers to coordinating our actions and thoughts, such as those reflected in Stroop and flanker tasks. The roles of attentional modulation across perceptual inputs and executive control of attention have been discussed in different sports [for a review, see Memmert (2009)]. In shooting and archery sports, the roles of arousal, vigilance, orienting, and conflict control have been stressed (Bertollo et al., 2012; Kim et al., 2019; Lu et al., 2021). However, the fact that many athletes of shooting and archery are actually shortsighted reminds us that there may also be some other attention ability that contributes to the success in shooting and archery. For example, Yifu Wang, a champion of 10-m air pistol in 1992 Barcelona and 2004 Athens Olympics, whose uncorrected vision is only 0.1. Short sight makes it difficult for those athletes to rely solely on visual information to aim the target, so their internal states may also be used. Such cases indicate that attention toward the self-status (specifically, interoceptive attention, Farb et al., 2013; Wang et al., 2019) may also be important to athletes of shooting and archery. This inference is consistent with the self-paced and far-aiming features of these two sports. As Moran (2004) noted, “the structure of a sport can affect its psychological requirements.” While some open skill sports (e.g., soccer and basketball) are physical-contact teamwork games with moving targets and may rely on visual attention, some closed skill sports, such as shooting and archery, are characterized by self-paced and far-aiming or targeting and thus may rely heavily on the status of athletes themselves. In shooting and archery sports, actions are mainly carried out at his/her own speed and without interference from others. This relatively isolated situation makes attention toward the self-status extremely important to achieve high performance and to become an elite athlete.

When considering attention toward the self-status, people may first consider proprioception. In fact, earlier research has found that there was a smaller body sway range in elite archers (Simsek et al., 2019) and that skilled air-pistol shooting players coordinated posture and upper-limb movements better than novice players (Ko et al., 2017). However, according to self-focus theories, when athletes perform automated skilled behaviors, directing their attention internally to themselves (i.e., focusing on task-related movements) would disrupt the automatic movements and thereby impair the performance (Masters and Maxwell, 2008). The body sway and coordination of limb movements, suggested by Simsek et al. (2019) and Ko et al. (2017), are the objective measures during shooting processes, rather than the focus of attention. It is likely that directing attention to proprioception may disrupt the automatic movement processes and exert negative influence on shooting and archery performances. Thus, we believed that attention to proprioception is not the self-status-oriented attention ability that acts as a positive contributor to an elite athlete.

If it was not attention to proprioception, then what is it that makes an elite shooter or archer? We proposed that interoceptive attention is important for an elite athlete in shooting and archery sports. Interoceptive attention represents a system associated with internal feelings of vasomotor activity, heartbeat, thirst, hunger, temperature, and other visceral sensations (Craig, 2002, 2003, 2010; Brener and Ring, 2016). The lamina I spinothalamocortical pathway, proposed by Craig (2002, 2003, 2010), provides a homeostatic afferent system that directly passes sensory signals of different modalities to the forebrain. The signals that represent physiological conditions of the body first input to lamina I and the nucleus of the solitary tract. Then, they are relayed by parabrachial nucleus, the medial dorsal nucleus and thalamus (i.e., the posterior ventral medial nucleus and the basal ventral medial nucleus) to the anterior cingulate cortex, insula, and other interoceptive cortex. The distinct feelings from the body appear as a result of the primary interoceptive representation in the dorsal posterior insula. Finally, these signals are transmitted into the right anterior insula where an ultimate meta-representation of the primary interoceptive activities emerges.

Under this lamina I spinothalamocortical pathway framework, an advantage in interoceptive attention may benefit athletes of shooting and archery in two ways. First, the representation of physiological conditions of the body (e.g., pain, temperature, muscular and visceral sensations, and vasomotor activity) may help the coordination of limb movements in aiming processes. Since the focus of attention is not the movement itself, interoceptive attention would not interrupt the automatic process of movements. In fact, when training athletes to pay attention to bodily signals such as heartbeat and respiratory signals, better performances were observed in running (Moran, 2000) and swimming (Couture et al., 1999). These results imply that interoceptive attention may be important for expert performance in sports. Second, it is believed that the integration representation of physiological condition of all tissues of the body underlies emotional awareness (Cannon, 1987; Damasio, 1996; Dolan, 2002; Craig, 2003; Critchley and Harrison, 2013). Sports performances are often impaired by anxiety of competition (Payne et al., 2019). Shooting and archery sports are no exception. Appropriate attention to physical state and accurate perception of internal sensory information are vital to maintain a normal physiological state and awareness of emotion (Craig, 2003, 2010; Critchley, 2005; Wiens, 2005). It is possible that shooters and archers with high interoceptive attention can accurately perceive the internal sensory signals and form an appropriate representation of physiological conditions of the body. This would enable athletes to avoid inappropriate emotions like anxiety that would negatively influence performance and would also help them perform better emotion regulation if inappropriate emotions arise. Therefore, we believed that interoceptive attention is important for shooting and archery, and there can be an advantage in interoceptive attention ability among elite athletes. The first aim of this study was to examine this hypothesis by testing the difference between elite and non-elite athletes of shooting and archery in interoceptive attention ability.

We used the breath detection task (BDT), suggested by Wang et al. (2019), as the interoceptive attention measurement in this study. In BDT, participants are asked to judge whether the respiratory curve displayed on screen is synchronized or delayed when compared with their own breathing rhythm. The control exteroceptive attention task asked participants to judge whether a red dot rapidly appeared on the respiratory curve (i.e., dot flash detection task, DDT). The accuracies of these two tasks represent the interoceptive and exteroceptive attention abilities of the individuals. By using BDT and DDT, Wang et al. (2019) demonstrated the involvement of the anterior insular cortex (AIC) in interoceptive attention. This finding appears to support the notion that an ultimate meta-representation of interoception forms in AIC (Craig, 2010; Critchley and Harrison, 2013). According to Garfinkel et al. (2016), there are three dimensions of interoception, namely, accuracy, sensibility, and metacognitive awareness. Earlier studies have proposed that accurate perceptions of physiological conditions of the body are important for emotion awareness (Critchley, 2005; Wiens, 2005; Craig, 2010). The association of reduced anxiety with greater accuracy of respiration task, suggested by Garfinkel et al. (2016), supported this perspective. Therefore, we focused on accuracy in this study. We chose the respiratory axis of interoception because compared with other axes (e.g., cardiac and stomach), respiration may be under greater voluntary control (Garfinkel et al., 2016). The availability of voluntary control may be important for implementing effective interventions. By comparing the difference in the performance of BDT and DDT between elite and non-elite athletes, we intended to improve our understanding of the relationship between expertise level and interoceptive attention in sports that are characterized by self-paced and far-aiming (e.g., shooting and archery).

If there is a relationship between expertise level and interoceptive attention ability, it preliminarily suggests that interoceptive attention is important for shooters and archers. Then, an important question is how to improve it. Recent research suggests that a promising candidate is mindfulness training. Mindfulness refers to “the awareness that emerges through paying attention on purpose in the present moment, and non-judgmentally to the unfolding of experience moment by moment” (Kabat-Zinn, 2003). Recent studies have found that mindfulness training significantly improved various attention abilities (He and Wang, 2020), such as sustained attention (MacLean et al., 2010; Jha et al., 2015; Bardart et al., 2018), conflict control (Elliott et al., 2014; Becerra et al., 2017), and selective attention distribution (Colzato et al., 2015; Schofield et al., 2015). In particular, a sport-specific mindfulness training program was applied to elite shooting athletes, and attention improvement was found after training (Bu et al., 2019). Since the mindfulness training program, such as mindfulness-based stress reduction (MBSR; Kabat-Zinn, 1982, 1990) and mindfulness-based cognitive therapy (MBCT; Teasdale et al., 1995, 2000), contains mindfulness of breath, sweeping, and other perceptions of the body, we believed that interoceptive attention can also be improved after training. Thus, the second aim of this study was to examine whether interoceptive attention can be improved by mindfulness training.

The self-paced and far-aiming features of shooting and archery and the fact that many athletes of shooting and archery are shortsighted reminded us that interoceptive attention ability supported by the lamina I spinothalamocortical pathway may also be important for success in these two sports. This study aims to examine the relationship between expertise level and interoceptive attention ability by testing if there is an advantage of elite shooters and archers in BDT, in comparison with non-elite athletes, and to investigate whether mindfulness training can improve interoceptive attention. We compared the performances of athletes from national and provincial teams in BDT and DDT. We predicted that athletes from the national team would outperform their counterparts from the provincial team in BDT. We also had elite shooters and archers in the national team who would receive mindfulness training for the time course of 5–8 weeks. Our hypothesis is that the interoceptive attention of elite athletes of shooting and archery will be improved after training.

## Materials and Methods

### Participants

Eighty-four athletes of shooting and archery participated in this study (46 females and 38 males, age: 21.73 ± 5.07 years, Min_age_ = 14, Max_age_ = 40). Forty-one of them (21 females and 20 males, age: 24.54 ± 5.38 years, Min_age_ = 16, Max_age_ = 40) were from the Chinese national team of shooting and archery, and the remaining 43 (17 females and 26 males, age: 19.05±2.85 years, Min_age_ = 14, Max_age_ = 29) were from the team of Hebei Province, China. We identified the athletes in the national team as the elite group because those athletes are top performers across the country and have been selected as candidates for international competitions. Informed consent was obtained from all athletes before the experiment. This study was approved by the Committee for Protecting Human and Animal Subjects, School of Psychological and Cognitive Sciences, Peking University, and it was conducted in accordance with the principles of the Declaration of Helsinki (World Medical Association, 2013).

### Procedure

All of the athletes participated in the BDT and DDT tests. Athletes in the national team then received mindfulness training for 5–8 weeks and took the BDT and DDT tests again. The order of BDT and DDT was counterbalanced in athletes. All athletes completed BDT and DDT in a quiet room. They sat in front of the testing computer in a comfortable position and were required to breathe in a normal and natural way during the whole experiment.

**Figure 1:**
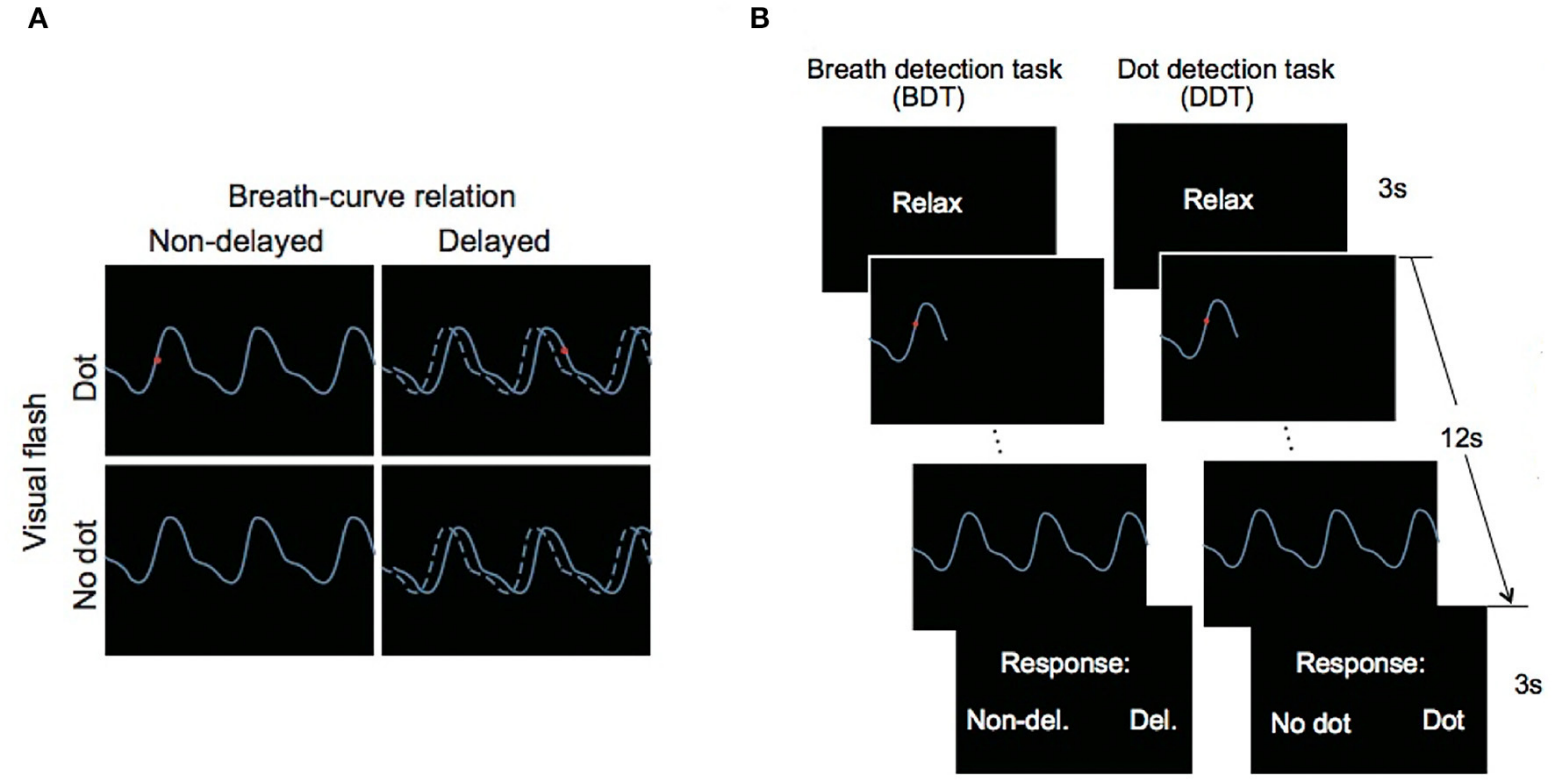
**(A)** Possible conditions. The solid line represents the respiratory curve displayed on the screen. The dashed line represents the actual breathing curve. **(B)** Sample of a trial in the breath detection task (BDT) and the dot flash detection task (DDT). Adapted from Wang et al. (2019).

The breath detection task (BDT) was designed to measure interoceptive attention, and DDT was designed to measure exteroceptive attention (Figure 1, Wang et al., 2019). The clear perception and autonomous control features of breathing make it feasible to measure interoceptive attention. The findings, suggested by Wang et al. (2019), which object interoceptive accuracy of BDT positively correlated with subjective rating of difficulty of interoceptive task has supported that BDT is a valid measurement of the interoceptive attention. According to Wang et al. (2019), the split-half reliabilities of both BDT and DDT in one sample are 0.86 and 0.85.

In BDT, breathing of the participants was converted into electronic signals by using the A/D converter (USB-1208HS-4AO, Measurement Computing, Inc., Norton, MA, USA), making recordings of their breathing as respiratory curves displayed on the screen. In each trial, after a 3-s relax screen, a respiratory curve would appear on the screen running from left edge to right edge for 12 s. The respiratory curve would be either real-time (non-delayed condition) or 0.4 s delay (delayed condition) to the breathing rhythm of the participants. Then, a response screen appeared for 3 s on which participants had to respond by choosing from two options to indicate whether the respiratory curve was synchronous or delayed. The interval between trials is 2 s. The procedure in DDT was similar to that in BDT, except that in half of the trials, a red dot would flash on the curve for 30 ms (dot condition) anytime during a 12 s period, and in the other half, no dot has appeared (no-dot condition). The task of the participants was to determine whether the red dot appeared. In the beginning, four illustration trials were administrated to help athletes get familiar with each task. The formal experiment began after eight practice trials. There are 40 trials in the formal experiment of each task (BDT and DDT) and 20 trials for each condition. It took about 50 min to finish these two tests.

The breathing of the athletes was recorded by using a respiratory transducer (TSD201, BIOPAC Systems Inc.) that was fastened around their upper chest to measure the thoracic circumference changes. Signals of circumference changes were sampled at 1,000 Hz by using the BIOPAC MP150/RSP100C system and passed through a DC amplifier with gain set to 10 V. The low-pass filtering was set at 1 Hz and high-pass filtering at 0.05 Hz. The digitalization of analog signals was finished by an A/D converter (USB-1208HS-4AO, Measurement Computing Inc., Norton, MA, USA), and the digitized signal was sent to a USB port of the test computer. The task program was run in E-Prime (Psychology Software Tools, Pittsburgh, PA, USA), which was used as an interface to present athletes the digitized signal from the USB port (indicated by continuous blue respiratory curves).

The mindfulness training comprised sweeping, mindfulness of breath, and other perceptions, which were adapted from MBSR (Kabat-Zinn, 1990) and MBCT (Teasdale et al., 2000). MBSR is an 8-week course of mindfulness training designed to help people achieve self-development, self-discovery, learning, and healing. MBCT is an intervention designed to help recovered recurrently depressed patients avoid relapse or recurrence. Some of the practices in MBCT were designed for depression, thus inappropriate for the present sample, so these practices were replaced by some content in MBSR (e.g., mindfulness yoga). In summary, the mindfulness training content included body scans, sitting meditation, walking meditation, and mindfulness yoga. Body scans referred to concentrating on the body sensation from head to toe. Sitting meditation referred to attending to and experiencing breath or thought when one is sitting in a comfortable position. Walking meditation referred to observing and sensing the moving parts of the body. Mindfulness yoga referred to focusing and keeping on stretching. Athletes of shooting and archery followed a professional coach to perform mindfulness training once a week for 1.5 h each time. They were asked to conduct a homework practice every day whenever they had time for 15 min.

### Data Analysis

The SPSS software version 21.0 (IBM, Armonk, NY, USA) was used for the data analysis. Performance accuracy (%) and discrimination sensitivity (*d*′) were calculated for BDT and DDT, respectively. We, first, compared performances of athletes with chance level by one-sample *t*-tests for both groups. Then, 2 (Group: National vs. Provincial) × 2 (Task: BDT vs. DDT) ANOVAs of accuracy and *d*′ were conducted to investigate the difference of interoceptive attention and exteroception attention between elite athletes from the national team and non-elite athletes in the provincial team. Finally, 2 (Time: Pre vs. Post) × 2 (Task: BDT vs. DDT) repeated-measure ANOVAs of accuracy and *d*′ were conducted to examine the effect of mindfulness training on interoceptive and exteroceptive attention in elite athletes. In addition, we also conducted a similar ANOVA analysis to examine the effect of mindfulness training for elite shooters and archers separately.

## Results

Accuracy and *d*′ of two tasks in both teams were significantly above the chance level (50% and 0 for accuracy and *d*′, respectively). For accuracy, BDT_national_: *t*(40) = 12.987, *p* < 0.001, Cohen's *d* = 2.03; DDT_national_: *t*(40) = 24.406, *p* < 0.001, Cohen's *d* = 3.81; BDT_provincial_: *t*(42) = 7.502, *p* < 0.001, Cohen's *d* = 1.14; DDT_provincial_: *t*(42) = 32.286, *p* < 0.001, Cohen's *d* = 4.92. For *d*′, BDT_national_: *t*(40) = 10.138, *p* < 0.001, Cohen's *d* = 1.58; DDT_national_: *t*(40) = 18.474, *p* < 0.001, Cohen's *d* = 2.89; BDT_provincial_: *t*(42) = 6.554, *p* < 0.001, Cohen's *d* = 1.00; DDT_provincial_: *t*(42) = 22.848, *p* < 0.001, Cohen's *d* = 3.47. Accuracy and *d*′ of two tasks after mindfulness training in national team were also significantly above the chance level. For accuracy, BDT_national_: *t*(40) = 26.730, *p* < 0.001, Cohen's *d* = 4.17; DDT_national_: *t*(40) = 28.423, *p* < 0.001, Cohen's *d* = 4.44. For *d*′, BDT_national_: *t*(40) = 18.226, *p* < 0.001, Cohen's *d* = 2.85; DDT_national_: *t*(40) = 21.191, *p* < 0.001, Cohen's *d* = 3.30.

### Differences in Interoceptive Attention Between Groups

Results of 2 (Group: National vs. Provincial) × 2 (Task: BDT vs. DDT) ANOVAs for accuracy (Table 1) showed a group effect, *F*_(1, 82)_ = 7.835, *p* = 0.006, partial η^2^ = 0.087, Diff_national−*provincial*_ = 6.04%, 95% CI = [1.7, 10.3%] and a task effect, *F*_(1, 82)_ = 32.189, *p* < 0.001, partial η^2^ = 0.282, Diff_BDT−*DDT*_ = −13.12%, 95% CI = [−17.5, −8.4%]. The group × task interaction was also significant, *F*_(1, 82)_ = 5.773, *p* = 0.019, partial η^2^ = 0.066. The simple effect analysis indicated that accuracy difference reached significant level only in BDT between groups, Diff_BDT_ = 11.50%, 95% CI = [3.7, 19.4%], *p* = 0.004. No significant difference was found in DDT, Diff_DDT_ = 0.50%, 95% CI = [−3.6, 4.7%], *p* = 0.797.

**Table 1:** Accuracy (%) in BDT and DDT between the groups.

	**BDT**				**DDT**			
	***M (SD)***	***S***	***K***	***K–S***	***M (SD)***	***S***	***K***	***K–S***
National (*n* = 41)	83.59 (16.56)	−0.97	−0.05	1.06	91.07 (10.78)	−2.96	12.10	1.47^*^
Provincial (*n* = 43)	72.05 (19.27)	−0.18	−1.27	0.91	90.53 (8.23)	−1.89	4.33	1.43^*^
Difference	11.54^**^	–	–	–	0.54	–	–	–

*M, mean; SD, standard deviation; S, skewness; K, kurtosis; K–S, Kolmogorov–Smirnov test; BDT, breath detection task; DDT, dot flash detection task*.

* *p < 0.05*,

** *p < 0.01*.

Similar results were obtained by 2 (Group: National vs. Provincial) × 2 (Task: BDT vs. DDT) ANOVAs for *d*′ (Table 2): group effect, *F*_(1, 82)_ = 9.818, *p* = 0.002, partial η^2^ = 0.107, Diff_national−*provincial*_ = 0.676, 95% CI = [0.247, 1.105]; task effect, *F*_(1, 82)_ = 30.127, *p* < 0.001, partial η^2^ = 0.269, Diff_BDT−*DDT*_ = −1.246, 95% CI = [−1.697, −0.794]; group × task interaction, *F*_(1, 82)_ = 4.536, *p* = 0.036, partial η^2^ = 0.052. The simple effect analysis indicated that *d*′ difference reached significant level only in BDT between groups, Diff_BDT_ = 1.159, 95% CI = [0.420, 1.898], *p* = 0.002; Diff_DDT_ = 0.193, 95% CI = [−0.287, 0.672], *p* = 0.427.

**Table 2:** Discrimination sensitivity (*d*′) in BDT and DDT between the groups.

	**BDT**				**DDT**			
	***M* (*SD*)**	***S***	***K***	***K–S***	***M* (*SD*)**	***S***	***K***	***K–S***
National (*n* = 41)	2.80 (1.77)	−0.16	−1.27	0.75	3.56 (1.23)	−0.98	1.91	0.70
Provincial (*n* = 43)	1.64 (1.64)	0.56	−0.46	0.87	3.37 (0.97)	−0.31	0.49	0.93
Difference	1.16^**^	–	–	–	0.19	–	–	–

*M, mean; SD, standard deviation; S, skewness; K, kurtosis; K–S, Kolmogorov–Smirnov test; BDT, breath detection task; DDT, dot flash detection task*.

** *p < 0.01*.

The results remained significant after controlling for age except the task effects for both accuracy and *d*′.

### Effects of Mindfulness Training on Interoceptive Attention in the National Team

Results of 2 (Time: Pre vs. Post) × 2 (Task: BDT vs. DDT) ANOVAs for accuracy (Table 3) showed a time effect, *F*_(1, 40)_ = 8.636, *p* = 0.005, partial η^2^ = 0.178, Diff_pre−*post*_ = −4.27%, 95% CI = [−7.2, −1.3%] and a time × task interaction, *F*_(1, 40)_ = 9.575, *p* = 0.004, partial η^2^ = 0.193. The simple effect analysis indicated that accuracy difference reached significant level only in BDT before and after mindfulness training, Diff_BDT_ = −8.5%, *p* = 0.001, 95% CI = [−13.5, −3.6%]; Diff_DDT_ = 0, *p* = 1.000, 95% CI = [−2.9, 2.9%]. Task effect was not significant, *F*_(1, 40)_ = 1.792, *p* = 0.188, partial η^2^ = 0.043, Diff_BDT−*DDT*_ = −3.22%, 95% CI = [−8.1, 1.6%].

**Table 3:** Accuracy (%) in BDT and DDT before and after the mindfulness training in the national team (*n* = 41).

	**BDT**				**DDT**			
	***M* (*SD*)**	***S***	***K***	***K–S***	***M* (*SD*)**	***S***	***K***	***K–S***
Pre	83.59 (16.56)	−0.97	−0.05	1.06	91.07 (10.78)	−2.96	12.10	1.47^*^
Post	92.12 (10.09)	−2.38	6.84	2.02^**^	91.07 (9.25)	−3.38	15.20	1.70^**^
Difference	−8.53^***^	–	–	–	0.00	–	–	–

*M, mean; SD, standard deviation; S, skewness; K, kurtosis; K–S, Kolmogorov–Smirnov test; BDT, breath detection task; DDT, dot flash detection task*.

* *p < 0.05*,

** *p < 0.01*,

*** *p < 0.001*.

Results of 2 (Time: Pre vs. Post) × 2 (Task: BDT vs. DDT) ANOVAs for *d*′ (Table 4) revealed a time × task interaction, *F*_(1, 40)_ = 11.461, *p* = 0.002, partial η^2^ = 0.223. The simple effect analysis indicated that *d*′ difference reached significant level only in BDT before and after mindfulness training, Diff_BDT_ = −0.822, *p* = 0.003, 95% CI = [−1.344, −0.300]; Diff_DDT_ = 0.229, *p* = 0.254, 95% CI = [−0.171, 0.629]. Neither time effect nor task effect reached significant level, *F*_(1, 40)_ = 3.049, *p* = 0.088, partial η^2^ = 0.071, Diff_pre−*post*_ = −0.29, 95% CI = [−0.639, 0.047]; *F*_(1, 40)_ = 0.767, *p* = 0.386, partial η^2^ = 0.019, Diff_BDT−*DDT*_ = −0.23, 95% CI = [−0.784, 0.310], respectively.

**Table 4:** Discrimination sensitivity (*d*′) in BDT and DDT before and after the mindfulness training in the national team (*n* = 41).

	**BDT**				**DDT**			
	***M* (*SD*)**	***S***	***K***	***K–S***	***M* (*SD*)**	***S***	***K***	***K–S***
Pre	2.80 (1.77)	−0.16	−1.27	0.75	3.56 (1.23)	−0.98	1.91	0.70
Post	3.62 (1.27)	−0.80	0.37	0.93	3.33 (1.01)	−0.69	3.12	0.65
Difference	−0.82^**^	–	–	–	0.23	–	–	–

*M, mean; SD, standard deviation; S, skewness; K, kurtosis; K–S, Kolmogorov–Smirnov test; BDT, breath detection task; DDT, dot flash detection task*.

** *p < 0.01*.

### Effects of Mindfulness Training on Interoceptive Attention in Elite Shooting Athletes in the National Team

Results of 2 (Time: Pre vs. Post) × 2 (Task: BDT vs. DDT) ANOVAs for accuracy (Table 5) showed a time effect, *F*_(1, 26)_ = 14.856, *p* = 0.001, partial η^2^ = 0.364, Diff_pre−*post*_ = −7.0%, 95% CI = [−10.7%, −3.3%], and a time × task interaction, *F*_(1, 26)_ = 5.619, *p* = 0.025, partial η^2^ = 0.178. The simple effect analysis indicated that accuracy difference reached significant level only in BDT before and after mindfulness training, Diff_BDT_ = −11.2%, *p* = 0.002, 95% CI = [−17.9, −4.6%]; Diff_DDT_ = −2.8%, *p* = 0.092, 95% CI = [−6.0, 0.5%]. Task effect was not significant, *F*_(1, 26)_ = 1.726, *p* = 0.200, partial η^2^ = 0.062, Diff_BDT−*DDT*_ = −4.5%, 95% CI = [−11.6, 2.6%].

**Table 5:** Accuracy (%) in BDT and DDT before and after the mindfulness training in elite shooting athletes of the national team (*n* = 27).

	**BDT**				**DDT**			
	***M* (*SD*)**	***S***	***K***	***K–S***	***M* (*SD*)**	***S***	***K***	***K–S***
Pre	80.30 (17.56)	−0.76	−0.46	0.84	89.04 (12.55)	−2.49	8.41	0.99
Post	91.52 (11.41)	−2.25	5.83	1.71^**^	91.81 (10.00)	−4.21	19.97	1.69^**^
Difference	−11.22^**^	–	–	–	−2.77	–	–	–

*M, mean; SD, standard deviation; S, skewness; K, kurtosis; K–S, Kolmogorov–Smirnov test; BDT, breath detection task; DDT, dot flash detection task*.

** *p < 0.01*.

Results of 2 (Time: Pre vs. Post) × 2 (Task: BDT vs. DDT) ANOVAs for *d*′ (Table 6) revealed a time effect, *F*_(1, 26)_ = 7.832, *p* = 0.010, partial η^2^ = 0.232, Diff_pre−*post*_ = −0.591, 95% CI = [−1.025, −0.157], and a time × task interaction, *F*_(1, 26)_ = 8.863, *p* = 0.006, partial η^2^ = 0.254. The simple effect analysis indicated that *d*′ difference reached significant level only in BDT before and after mindfulness training, Diff_BDT_ = −1.163, *p* = 0.001, 95% CI = [−1.828, −0.498]; Diff_DDT_ = −0.019, *p* = 0.937, 95% CI = [−0.515, 0.477]. The task effect did not reach significant level, *F*_(1, 26)_ = 0.728, *p* = 0.401, partial η^2^ = 0.027, Diff_BDT−*DDT*_ = −0.315, 95% CI = [−1.075, 0.444].

**Table 6:** Discrimination sensitivity (*d*′) of BDT and DDT before and after the mindfulness training in elite shooting athletes of the national team (*n* = 27).

	**BDT**				**DDT**			
	***M* (*SD*)**	***S***	***K***	***K–S***	***M* (*SD*)**	***S***	***K***	***K–S***
Pre	2.46 (1.77)	0.09	−1.21	0.57	3.35 (1.36)	−0.81	1.25	0.62
Post	3.62 (1.35)	−0.96	0.53	0.98	3.37 (1.03)	−1.43	5.20	0.97
Difference	−1.16^***^	–	–	–	−0.02	–	–	–

*M, mean; SD, standard deviation; S, skewness; K, kurtosis; K–S, Kolmogorov–Smirnov test; BDT, breath detection task; DDT, dot flash detection task*.

*** *p < 0.001*.

### Effects of Mindfulness Training on Interoceptive Attention in Elite Archery Athletes in the National Team

Results of 2 (Time: Pre vs. Post) × 2 (Task: BDT vs. DDT) ANOVAs for accuracy (Table 7) revealed no significant effects. Time effect: *F*_(1, 13)_ = 0.329, *p* = 0.576, partial η^2^ = 0.025, Diff_pre−*post*_ = −1.0%, 95% CI = [−2.8%, 4.8%]. Task effect: *F*_(1, 13)_ = 0.088, *p* = 0.771, partial η^2^ = 0.007, Diff_BDT−*DDT*_ = −0.07%, 95% CI = [−5.9%, 4.5%]. Time × task interaction: *F*_(1, 13)_ = 3.868, *p* = 0.071, partial η^2^ = 0.229.

**Table 7:** Accuracy (%) of BDT and DDT before and after the mindfulness training in elite archery athletes of the national team (*n* = 14).

	**BDT**				**DDT**			
	***M* (*SD*)**	***S***	***K***	***K–S***	***M* (*SD*)**	***S***	***K***	***K–S***
Pre	89.93 (12.71)	−1.40	0.87	1.16	95.00 (4.15)	−0.94	1.35	0.64
Post	93.29 (7.13)	−2.18	6.23	1.01	89.64 (7.75)	−0.57	−0.46	0.63
Difference	−3.36	–	–	–	5.36	–	–	–

*M, mean; SD, standard deviation; S, skewness; K, kurtosis; K–S, Kolmogorov–Smirnov test; BDT, breath detection task; DDT, dot flash detection task*.

Results of 2 (Time: Pre vs. Post) × 2 (Task: BDT vs. DDT) ANOVAs for *d*′ (Table 8) revealed no significant effects. Time effect: *F*_(1, 13)_ = 1.482, *p* = 0.245, partial η^2^ = 0.102, Diff_pre−*post*_ = 0.272, 95% CI = [−0.211, 0.754]. Task effect: *F*_(1, 13)_ = 0.056, *p* = 0.816, partial η^2^ = 0.004, Diff_BDT−*DDT*_ = −0.086, 95% CI = [−0.863, 0.692]. Time × task interaction, *F*_(1, 13)_ = 2.576, *p* = 0.133, partial η^2^ = 0.165.

**Table 8:** Discrimination sensitivity (*d*′) of BDT and DDT before and after the mindfulness training in elite archery athletes of the national team (*n* = 14).

	**BDT**				**DDT**			
	***M* (*SD*)**	***S***	***K***	***K–S***	***M* (*SD*)**	***S***	***K***	***K–S***
Pre	3.44 (1.63)	−0.67	−0.81	0.69	3.96 (0.83)	−0.28	0.75	0.62
Post	3.61 (1.14)	−0.32	0.04	0.47	3.26 (1.01)	1.03	0.14	0.77
Difference	−0.17	–	–	–	0.70	–	–	–

*M, mean; SD, standard deviation; S, skewness; K, kurtosis; K–S, Kolmogorov–Smirnov test; BDT, breath detection task; DDT, dot flash detection task*.

## Discussion

This study demonstrated the relationship between expertise level and interoceptive attention and showed that mindfulness training can effectively improve interoceptive attention in elite athletes of shooting and archery. These results provided preliminary evidence for the importance of interoceptive attention in shooting and archery sports.

Comparisons of performances between the national and provincial groups showed that elite shooters and archers outperform athletes from provincial teams in BDT, whereas the two groups did not significantly differ from each other in DDT. A similar pattern was found in *d*′ results which showed that the sensitivity of elite shooters and archers was higher than that of provincial athletes in BDT, but not in DDT. These results suggest that elite shooters and archers indeed have higher interoceptive attention ability. In other words, they can better perceive the somatic and visceral responses or changes and discriminate these signals from noises. According to Critchley and Harrison (2013), the afferent input of the internal body signals serves as sources on which individuals organize cognitive, emotional, and behavioral responses. This may be the mechanism through which the advantage of elite athletes in interoceptive attention exerts its influence. Higher ability in interoceptive attention may be important in the preparation stage during which athletes can adjust their postures based on the current physiological states. It may also benefit the regulation of negative emotions, given the notion that the arising of emotions is inseparable from somatic reactions and visceral signals (Critchley, 2005; Wiens, 2005; Craig, 2010). During the competition, once negative emotion like anxiety arises, accurately perceiving the accompanied physiological changes such as increased heartbeat and muscle stiffness may be the first step to adjustment and further regulation of negative emotions.

The results of mindfulness training showed that the performance of elite shooters and archers improved significantly in BDT but remained unchanged in DDT after receiving mindfulness training. The sensitivity in BDT, but not in DDT, also improved after mindfulness training. Even though elite shooters and archers showed better performance in BDT than provincial players, mindfulness training still improved their interoceptive attention ability. This improvement may benefit from the content of mindfulness training. The content includes keeping focus on a particular point or experience, such as sweeping and mindfulness of breath in a non-judging way (Kabat-Zinn, 1982). Athletes were instructed to redirect their focus to the current attention (e.g., sweeping and breathing) whenever they found their mind drifting somewhere else. Through continuous practice, they were eventually able to keep their attention on bodies for a long period of time, allowing them to quickly and accurately detect physiological changes. We speculated that the neural mechanism that supports this improvement may rely on AIC. On the one hand, the lamina I spinothalamocortical pathway hypothesis indicates that AIC is the destination of transmission of signals that represent physiological conditions of the body. The right AIC integrates the afferent signals and generates an ultimate meta-representation of the primary interoceptive activities (Craig, 2002, 2003, 2010). The finding of AIC activity related to interoceptive attention, suggested by Wang et al. (2019), supports this hypothesis. On the other hand, studies on mindfulness training consistently report increased activity (Lutz et al., 2014; Wheeler et al., 2017; Young et al., 2018), gray matter thickness (Lazar et al., 2005), and gyrification (Luders et al., 2012) of the AIC. So, it may be possible that the mindfulness training increases AIC activity, gray matter thickness, gyrification, or all of them in the elite athletes, and these increased characteristics of AIC lead to accurate perception of bodily states, which manifest in higher performance of BDT. This may also explain why there was no improvement in the performance of DDT. However, the result that no improvement was found in the performance of DDT does not lead to the conclusion that mindfulness training has no effect on exteroceptive attention. The sensory input of exteroceptive attention has different modalities. We used visual stimulus in this study. Future studies can examine the effect of mindfulness training on other modalities of exteroceptive attention (e.g., auditory stimuli). There are possibilities that mindfulness training may influence the attentional modulation of different perceptual and sensory inputs.

Studies have demonstrated that 4 weeks of mindfulness training could have an influence on dimensions of trait variables in the athletes (Kaufman et al., 2009). Although we initially planned an 8-week training period, due to the different training schedules of elite athletes, it is hard to guarantee the full training period of each athlete. But we managed that all athletes received at least 5 weeks of mindfulness training. A recent review on mindfulness using meta-analysis concluded that a course of 5 weeks would be sufficient to influence the trait mindfulness of an individual (Buhlmayer et al., 2017). Thus, we believed that mindfulness training in this study did exert influence. But it is possible that the additional 3-week training further improved some performance of the athlete compared with the 5-week training. This indicates that our results may reflect a moderate effect of mindfulness training on interoceptive attention. Future studies are recommended to explore the relationship of mindfulness training period and its effect on interoceptive attention in shooting and archery. Some may raise concerns that our experimental design cannot rule out the possibility of practice effect since there was no control group. Should it be a mere practice effect, the performance of the athletes in both BDT and DDT should have been improved. Instead, the results of this study showed an enhancement only in BDT. These results suggest that the improvement of interoceptive attention was not merely a practice effect, but resulted from mindfulness training. Follow-up studies can further investigate the effect of mindfulness training on interoceptive attention in athletes of shooting and archery through random sampling or by adding a control group that does not receive mindfulness training or with brain imaging techniques such as fMRI.

We admitted that there are limitations in this study, and future research can further investigate the role of interoceptive attention in shooting and archery sports in the following aspects. First, our results provide preliminary evidence for the relationship between expertise level and interoceptive attention. But the causal relationship cannot be specified because we did not measure shooting and archery performances. It is possible that the advantage in interoceptive attention results from the high expertise level of elite athletes. Future studies can improve our knowledge about this issue by measuring performances of shooting and archery simultaneously in longitudinal designs. Studies have shown that mindfulness training can improve shooting competition performances (Solberg et al., 1996; John et al., 2011) and facilitates emotion regulation (Wheeler et al., 2017). We speculated that there may be the mediation effects of interoceptive attention and emotion on mindfulness training improving shooting and archery performances. Future studies are encouraged to test these hypotheses by monitoring performances, interoceptive attention during mindfulness training.

Second, the mindfulness training in this study was part of a Psychology Service Program of General Administration of Sport of China for the national team of shooting and archery, thus were only offered to elite athletes. Although it would be more rigorous if the non-elite athletes (provincial team) also received mindfulness training, we believed that the study of mindfulness training in elite athletes also benefits the literature. Our results showed that even though elite athletes had an advantage in interoceptive attention, they still had the possibility of further improvement. We speculated that the effect of mindfulness training has also to be observed in non-elite athletes, and we hope that the findings in this study are also helpful for non-elite athletes. Future studies are welcomed to investigate the effect of mindfulness training on athletes with different skill levels.

Third, in this study, we focused on athlete population only. The comparison between elite and non-elite athletes will give its own contribution to the field. The experiment with non-athlete would further justify the importance of interoceptive attention for elite shooters and archers. We admit that an inclusion of a control group in the mindfulness training experimental design would be more rigorous. Though researchers have found that gender can influence the mindfulness training effect on emotion regulation (Brown et al., 2020), the fact that there was no gender ratio difference between the national and provincial teams relieved us from the suspicion of gender difference confounding. Future studies are still welcomed to explore how gender moderates the effect of mindfulness training in sports of shooting and archery.

On the topic of relationship between interoceptive attention and sports of shooting and archery, this study suggests at least two directions for future research. First, we identified the advantage and improvement of elite athletes in interoceptive attention and proposed its possible influence in shooters and archers, but it remains unknown how the interoceptive attention helps athletes in performance. In addition to better emotional regulation, another possibility is that interoceptive attention may facilitate the formation of automatic movements. The follow-up studies can explore the influence of interoceptive attention on the formation and implementation of automatic movements that are believed important for excellent performance. These can be done by comparing the differences between elite and novice athletes in the longitudinal research. Furthermore, in addition to shooting and archery, future research can also investigate the effect of interoceptive attention on other self-paced and far-aiming sports, such as golf. Interoceptive attention may exert a similar influence on golfers to achieve excellent performances.

Second, at present, in order to help athletes of shooting and archery better perceive their own physiological state during daily training, biofeedback devices are used (Mullineaux et al., 2012; Ortega and Keng, 2018). The biofeedback instrument amplifies the physiological signals to the extent at which they are visually salient to help athletes adjust their coordination of body parts. However, in official competitions, athletes are not allowed to bring the biofeedback equipment on the field. Whether the athletes can still accurately perceive their own bodily signals or responses and make timely adjustments without the aid of the instrument remains to be seen. According to our results, mindfulness training can improve the interoceptive attention of athletes without using external equipment that amplifies the signals. Subsequent research can test whether the combination of biofeedback and mindfulness training may further assist athletes of shooting and archery.

## Conclusion

In this study, we found that when compared with athletes in the provincial team, the elite athletes in the national team performed better in BDT, but not in DDT. This result demonstrated the relationship between expertise level and interoceptive attention ability. Moreover, we found that mindfulness training could improve the BDT performance in elite athletes of shooting and archery. These results intensified our understanding of the role of interoceptive attention in self-paced far-aiming sports and have important implications for the selection and training of shooting and archery athletes. Interoceptive attention may be one of the key cognitive abilities for an elite shooter or archer. From the results, we suggested that mindfulness practice can be included in the training of athletes of shooting and archery.

## Data Availability Statement

The raw data supporting the conclusions of this article will be made available by the authors, without undue reservation.

## Ethics Statement

The studies involving human participants were reviewed and approved by the Committee for Protecting Human and Animal Subjects, School of Psychological and Cognitive Sciences, Peking University. Informed consent to participate in this study was provided by the participants' legal guardian/next of kin.

## Author Contributions

PL contributed to the experimental design, data collection and analysis, and drafting of the manuscript. QL contributed to the experimental design and data collection. QW contributed to the experimental design, data analysis, and revision of the manuscript. XL contributed to the data collection and examined the study. YW conceived of and examined the study, and revised the manuscript. All authors contributed to the article and approved the submitted version.

## Conflict of Interest

The authors declare that the research was conducted in the absence of any commercial or financial relationships that could be construed as a potential conflict of interest.
